# Measuring your dependence: deranged corticobulbar excitability may uncover addiction disorders

**DOI:** 10.3389/fpsyt.2012.00107

**Published:** 2012-12-12

**Authors:** Carmelo M. Vicario, Luca F. Ticini

**Affiliations:** ^1^University of Queensland, School of PsychologyBrisbane, Australia; ^2^Max Planck Institute for Human Cognitive and Brain SciencesLeipzig, Germany

Major efforts in neurobiology are currently directed toward the understanding of the neural mechanisms of reward (e.g., in food or monetary compensation). Neuroimaging studies have identified a broad network of brain areas involved in computing the rewarding value of stimuli, which comprises the orbitofrontal cortex (OFC), the anterior cingulate cortex (ACC), amygdala, insula, and several dopaminergic midbrain regions such as the ventral and dorsal striatum (for a review, see Kringelbach, 2005).

When these neural structures are malfunctional, addiction, impulsivity, and substance abuse can occur in human as well as in non-human mammals. For instance, obesity is associated with reduction of D2 receptors in the striatum and decreased metabolic activity in OFC and ACC (Trembaly and Schultz, 1999). Pathological gambling and alcoholism correlate with reduced ventral striatal activity and abnormalities in the left–right symmetry of the insular cortex (Jung et al., 2007). Along with these results, abnormal activation in the ventral striatum/nucleus accumbens in addicted smokers was demonstrated during presentation of smoking-related cues (David et al., 2005).

On the one hand, the importance of neuroimaging techniques (such as functional magnetic resonance imaging, fMRI) in localizing the brain correlates of addiction is well-established. However, blood oxygen level dependent fMRI alone does not determine whether neural activity is excitatory or inhibitory in nature, it has low temporal resolution and its signal represents the local field potentials, thus reflecting more the local processing of inputs rather than the output signals (Goense and Logothetis, 2008). Other methodological approaches, such as transcranial magnetic stimulation (TMS), may shed light on neglected aspects of addiction, by characterizing the abnormalities and physiological disturbances that lead to it. TMS has been employed in addicted patients to measure corticospinal excitability, an index of how much the activity of the corticospinal tract (projecting from the motor cortex to the spinal cord) is facilitated under given conditions (for a review, Feil and Zangen, 2010). These studies have mainly investigated chronic smokers. However, the fact that the excitability of the corticospinal tract is generally sensitive to substances such as nicotine (Thirugnanasambandam et al., 2011) casts some doubts on the possibility of employing this measure to detect states of addiction. We hereby propose that a more informative evaluation of this clinical condition might be obtained by measuring the excitability of the corticobulbar tract, which connects the motor cortex to the brainstem and the muscles thus innervated: those of the tongue, pharynx, and larynx. A recent hypothesis has suggested that in Anorexia Nervosa somatosensory intra-oral activity may reflect food reward alterations (Vicario and Candidi, 2011). Here, we propose that the assessment of corticobulbar excitability might be used to investigate the patophysiology of the reward system and of its temporal dynamics in addiction. In particular, in addicted patients the abnormal sensitivity to relevant stimuli (i.e., alcoholic drinks, cigarettes, and food) would be expressed as deranged excitability of the corticobulbar tract. Technically speaking, corticobulbar excitability can be measured by recording TMS-elicited motor-evoked potentials (MEP) from the tongue muscles. The discharge of a single magnetic pulse at the scalp surface in correspondence of the tongue representation in the primary motor cortex, induces electric fields causing neurons to depolarize. This allows to directly investigating excitability by quantifying the magnitude of the response represented by MEP. Differences in MEP amplitude correspond to differing levels of corticobulbar excitability and thus, we hypothesize, they could provide insights into the deranged activity of the reward system. Indeed, anatomo-functional evidences indicate that the tongue is probably the only somatotopic surface provided with receptors (for taste) which directly connects to those brain regions involved in estimating the reward properties of a stimulus, i.e., the frontal operculum, the anterior insula, and the primary taste cortex that projects anteriorly to the caudolateral orbitofrontal region (Ogawa, 1994). Of particular relevance for this proposal is also the observation that, in mice, chronic exposure to nicotine during embryonic development and during the first week of life alters neurotransmission and excitability in hypoglossal motoneurons projecting to the tongue (Pilarski et al., 2011).

We believe that this methodological approach should be considered in the context of translational and clinical psychiatry, having the potential to critically contribute to the understanding of addiction by informing us on pathophysiological aspects that other imaging methods currently don't provide.
